# 25-year follow-up of 102 unrevised cementless total hip arthroplasties with a second-generation metal-on-metal bearing in a young patient cohort

**DOI:** 10.1186/s42836-026-00408-4

**Published:** 2026-07-02

**Authors:** Moritz von Falkenhayn, Andre Lunz, Max Kempe, Timo A. Nees, Kevin-Arno Koch, Moritz M. Innmann, Tilman Walker, Tobias Reiner

**Affiliations:** Department of Orthopaedics, Heidelberg University Hospital, Schlierbacher Landstr. 200a, 69118 Heidelberg, Germany

**Keywords:** Hip, Total Hip Arthroplasty, Implant Survival, Metasul, Metal-on-Metal Bearings, Adverse Reactions to Metal Debris, ARMD

## Abstract

**Background:**

Second-generation metal-on-metal (MoM) bearings were reintroduced in total hip arthroplasty (THA) for their low wear rates, but despite initially promising results, concerns have emerged about adverse reactions to metal debris and poor long-term implant survival, with limited and conflicting evidence regarding the outcomes of small-diameter MoM implants. This study aimed to evaluate implant survival and metal wear complications in young patients with second-generation small-head MoM THA after more than 20 years.

**Methods:**

A retrospective cohort study was conducted at a university center, analyzing 179 consecutive THAs in 160 patients aged under 55 years, using a small-head MoM bearing. After a minimum 20-year follow-up, clinical-radiological outcomes and systemic metal ion concentrations were assessed, with Kaplan–Meier analysis determining implant survival rates.

**Results:**

Mean patient age at surgery was 46 years. After a mean follow-up of 23 years, 38 (21%) patients had deceased, and 9 (5%) patients were lost to follow-up. A total of 30 (16%) THAs had undergone revision surgery. The reasons for revision were metal wear-related complications in 11 hips (6%), aseptic loosening in 9 hips (5%), periprosthetic fractures in 6 hips (3%), periprosthetic joint infection in 3 hips (2%), and recurrent dislocation in 1 hip (1%). Of the non-revised THAs, the mean WOMAC score was 85.3. Osteolytic lesions were identified in 43% of hips with available radiographs (29/67), and elevated blood chromium or cobalt ion levels (> 2 µg/L) were detected in 22% of hips with available blood samples (11/51). Magnetic resonance imaging with metal artifact reduction sequence (MARS-MRI) was performed in 18 unrevised THAs, revealing a pseudotumor in 3 (17%). Kaplan–Meier survival rates at 25 years were 93% (95% CI: 87%–96%) for metal wear-related revisions and 77% (95% CI: 67%–85%) for any revision.

**Conclusions:**

Second-Generation small-head MoM THA leads to acceptable implant survival rates and joint function more than 20 years after index surgery. Still, metal wear-related complications were the most frequent cause for revision (37%), and periprosthetic osteolysis was present in 43% of hips. This highlights the need for long-term monitoring into the third decade. Considering the superior performance of modern bearings, their use is recommended to avoid wear-related complications and revisions.

## Background

Since conventional polyethylene inlays led to high wear in total hip arthroplasty (THA), second-generation metal-on-metal (MoM) bearings were reintroduced in the late 1980s [1]. Due to promising low volumetric wear rates, they were utilized especially in young and active patients with high demands. Well into the 2000s, MoM bearings were frequently utilized, with 11% of all uncemented hip replacements in the United Kingdom incorporating MoM articulations in 2008 [2].

Despite low volumetric wear rates of MoM bearings in simulator studies, some implants demonstrated poor clinical outcomes and high failure rates associated with adverse reactions to metal debris (ARMD) [3], particularly in cases involving large diameter femoral heads. Complications of MoM bearings encompass a spectrum of adverse outcomes, including pseudotumor formation, metallosis, and increased systemic metal ion exposure, most notably of cobalt and chromium [3–5].

Latest registry data have underscored the poor survival rates of MoM implants compared to alternative bearing surfaces in THA [2]. However, comprehensive long-term data on the performance of small-diameter MoM implants remains limited and inconclusive. Conflicting reports exist in the literature, with some studies indicating high rates of implant revision, while others report good mid- to long-term outcomes [6–8] As a consequence, clear consensus recommendations on how closely these patients need to be monitored and how to proceed if metal wear-associated complications are diagnosed in the long term are missing.

This study aims to (i) evaluate the clinical and radiological outcome of second generation small-head (28 mm) MoM cementless THAs at a minimum follow-up of 20 years in a young patient cohort, (ii) conduct implant survival analyses and determine the incidence of metal wear-associated complications and (iii) investigate blood metal ion concentrations of cobalt and chromium at long term follow-up among young and physically active patients.

## Methods

### Study design and patients

This is a retrospective, single-center, multi-surgeon cohort study. It includes a consecutive series of 160 patients (179 hips) following cementless THA. A Metasul (Zimmer Biomet, Warsaw, IN, USA) second-generation MoM articulation was implanted in all patients. This consists of a 28 mm diameter forged, high-carbide (0.2–0.25%) cobalt-chromium alloy femoral head and an acetabular liner, which is embedded in a polyethylene insert, consistent with a sandwich-type design. Furthermore, a cementless, press-fit titanium acetabular shell and an uncemented straight-tapered stem with a standard 12/14 mm Euro taper were used in all patients.

Approval was obtained by the ethics committee of the Heidelberg School of Medicine (S-365/2013), and each patient gave written consent before inclusion in the study. Surgery was performed consecutively between April 1995 and November 2001 at a single institution. A modified Watson-Jones or a transgluteal lateral approach was utilized in all patients. Indications for the use of a MoM bearing at this time were young patient age and a high anticipated physical activity level.

### Clinical and radiographic follow-up

Clinical examination and radiographic analysis were performed in a standardized fashion by a medical student and an orthopaedic surgeon. Additionally, the WOMAC score was utilized. Standard anteroposterior pelvis and lateral radiographs of the hip were evaluated regarding loosening, radiolucent lines, and osteolysis. Periprosthetic osteolysis was defined as a lucent zone absent of trabecular bone, which was not visible on the immediate postoperative radiograph [9]. Radiolucencies and osteolysis were evaluated conforming to the zones described by Gruen et al. [10] and the classification system of DeLee and Charnley [11]. The inclination angles of the cups were determined with the inter-teardrop line serving as a fixed landmark [12].

In addition, cross-sectional imaging with a metal artifact reduction sequence magnetic resonance imaging (MARS MRI) was indicated in patients with blood cobalt or chromium ion levels > 2 μg/L, previously documented pseudotumor formation, or high clinical suspicion for ARMD. MRI was evaluated regarding ARMD formation by at least one radiology consultant and one orthopaedic surgeon.

### Metal ion analysis

Blood samples were collected using a blood collection system designed specifically for trace metal ion analysis (Sarstedt, Nuembrecht, Germany; Refs. 58.1162.600 and 01.1604.400). Whole blood metal ion analysis for cobalt and chromium was conducted at the Limbach Laboratories, Heidelberg, using high-resolution, inductively coupled, plasma-mass spectrometry (ICP-MS). ICP-MS is considered one of the preferred techniques for measuring blood metal ion levels [13]. Using the same methodology, metal ion concentrations were measured as part of a study conducted ten years prior and used for comparison with current values [14]. A clinically relevant increase or decrease was defined as a change exceeding 1.0 μg/L.

### Statistical methods

Statistical analysis was conducted using SPSS for Windows (Version 29.0; IBM, Armonk, NY, USA) and Graphpad Prism (Version 9, GraphPad Software, San Diego, CA, USA). Descriptive statistics were conducted using the arithmetic mean, standard deviation, median, minimum, and maximum. Kaplan–Meier survivorship analysis was performed, with revision related to metal wear and revision for any reason as endpoints. A paired t-test was utilized to assess statistical significance concerning blood metal ion levels after 14.5 years and at the present follow-up. Statistical significance was considered with a *p*-value of < 0.05.

## Results

### Cohort

**Fig. 1:**
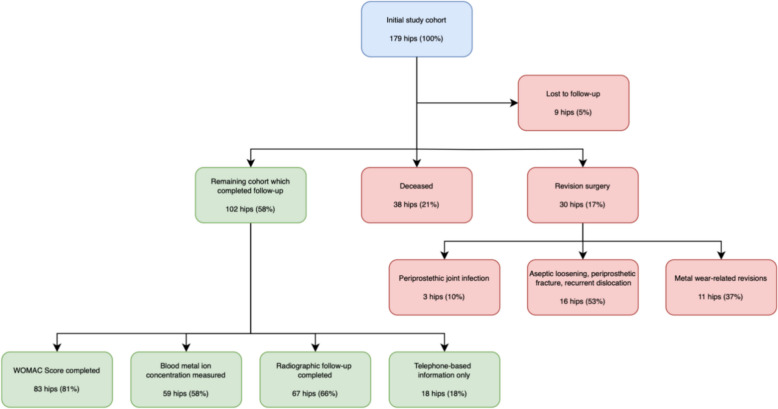
Flowchart depicting the follow-up status of the cohort

The mean age of patients at the time of the index surgery was 46 years (range: 21–55 years). At a mean follow-up of 23 years (range 20–28), 38 patients (21.2%) had died, and 9 (5.0%) were lost to follow-up. Based on telephone inquiries and oral statements from patients’ relatives, it has been ascertained that all deaths were unrelated to the THA, and no revision surgeries had been performed prior to the THA. Furthermore, 30 hips (16.8%) had undergone revision surgery at the time of the latest follow-up, leaving 102 unrevised hips available for follow-up and review. A radiographic follow-up was completed for 67 hips (65.7%). Blood metal ion analyses were conducted in 51 hips (50%). All other patients were contacted via mail and telephone to ensure no revision surgery had been performed nor any other complications had occurred (Fig. 1).

### Revisions

The causes for revision were documented as follows: recurrent dislocation in 1 patient (0.6%), periprosthetic joint infections in 3 patients (1.7%), periprosthetic fractures in 6 patients (3.4%), aseptic loosening in 9 patients (5%), and metal wear-associated complications, including significant pseudotumor formation and/or elevated systemic metal ion levels, in 11 patients (6%). In 6 of these 11 (54.5%) cases, an isolated revision of the head and liner was performed. In all cases, a ceramic head with a highly crosslinked polyethylene liner was implanted during revision surgery. Due to loosening or severe osteolysis, in 5 of these 11 (45.5%) cases, the cup and/or stem had to be revised. The timepoint of revision surgery for metal-wear-related complications varied, ranging from 7 to 26 years after implantation.

### Survival analysis

**Fig. 2:**
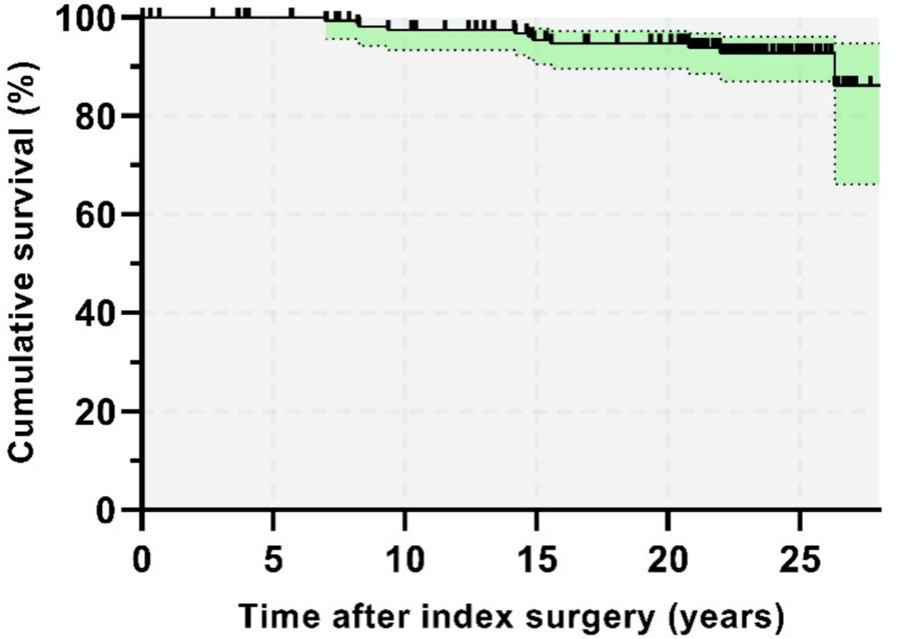
Kaplan–Meier survival for the endpoint “metal wear-related implant revision”

**Fig. 3:**
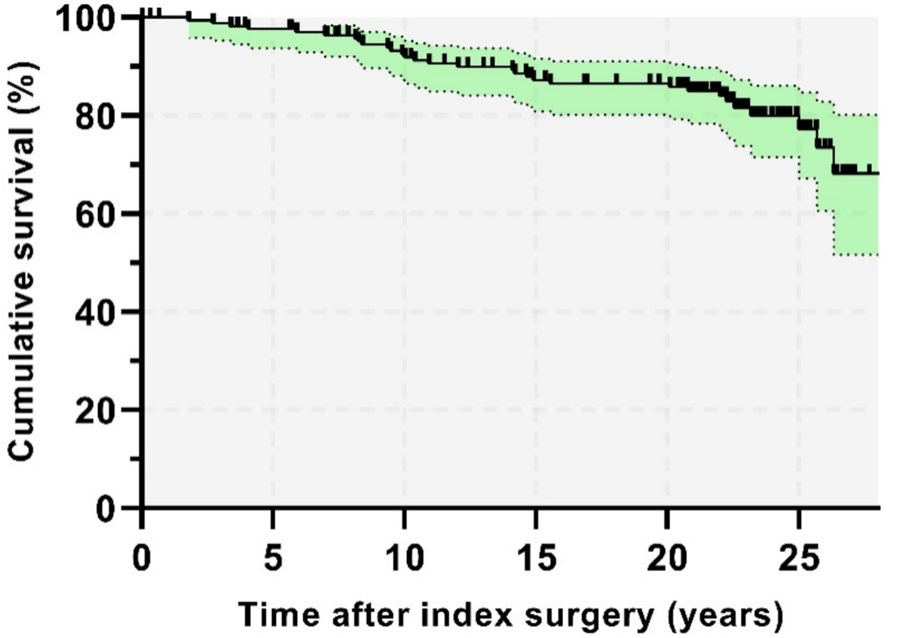
Kaplan–Meier survival for the endpoint “any implant revision”

Kaplan–Meier-survival analysis demonstrated a 10-, 20- and 25-year survival rate of 97% (95% CI: 93%–99%; 150 hips at risk), 95% (95% CI: 90%–97%; 129 hips at risk), and 93% (95% CI: 87%–96%; 30 hips at risk) for the endpoint “metal wear-related revision” (Fig. 2). The estimated 10-, 20- and 25-year survival for the endpoint “any revision” was 92% (95% CI: 87%–95%; 144 hips at risk), 86% (95% CI: 79%–90%; 120 hips at risk), and 77% (95% CI: 67%–85%; 30 hips at risk; Fig. 3).

### Clinical evaluation

The WOMAC score was available for 83 of 102 unrevised hips (81.4%). For improved comparability, the score was reversed and converted to a 100-point scale, with 0 indicating the poorest outcome and 100 representing the best possible result. At a mean follow-up of 23 years, the mean WOMAC score was 85.3 (range: 34–100).

### Radiographic evaluation

**Fig. 4:**
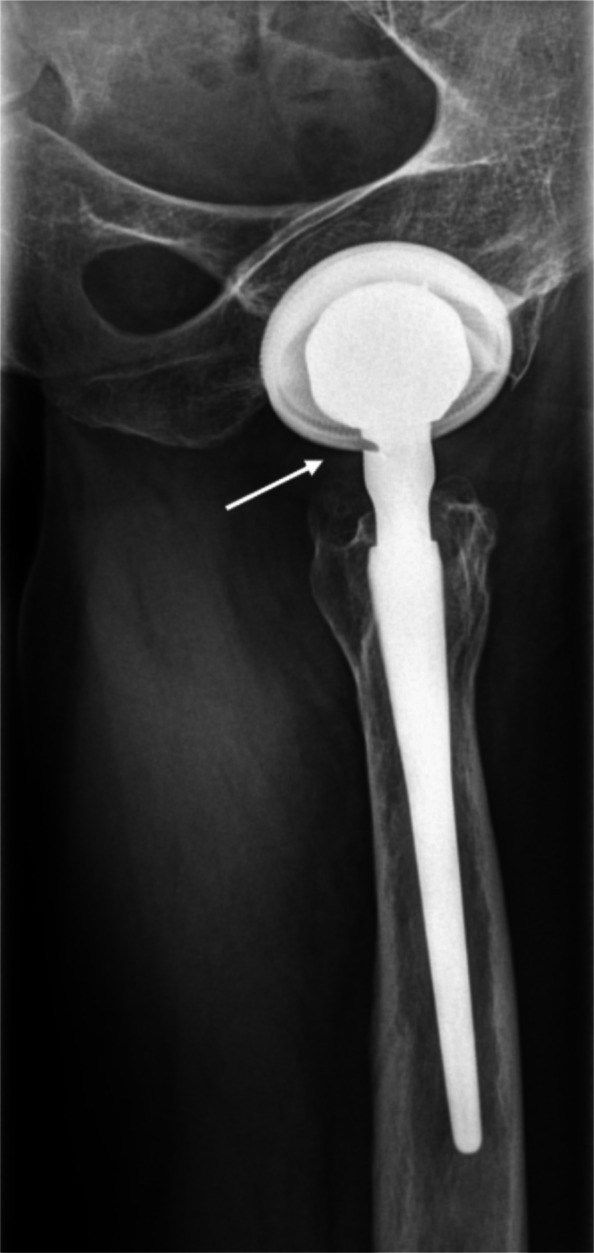
Axial radiograph of the left THA of a 60-year-old female patient, taken 25 years post index-surgery, demonstrating severe notching of the implant stem

After a minimum follow-up of 20 years, current radiographs were available for 67 out of 102 unrevised hips (65.7%). No hips showed any signs of cup or stem loosening. Mean acetabular inclination angle was 44.6° (range: 25–62°). An inclination angle > 50° was observed in 13 hips (19.4%). Asymptomatic minor osteolysis was exhibited in 29 of 67 hips with available radiographs (43.3%). Osteolysis and radiolucencies were documented mainly around the proximal Gruen zones 1 and 7 of the femoral components. Furthermore, intra-articular impingement, characterized by notching of the implant stem, was observed in 5 hips (7%) through conventional radiographic techniques (Fig. 4).

**Fig. 5:**
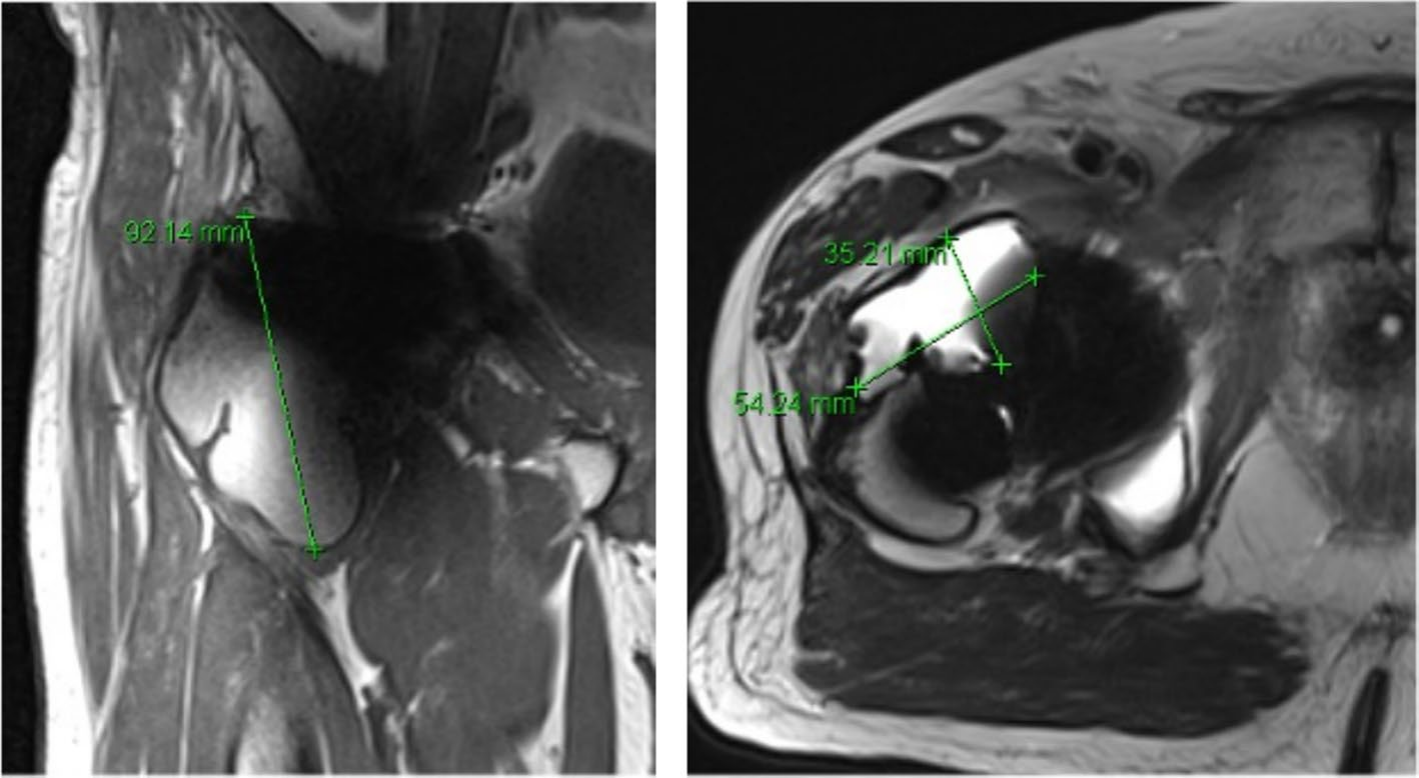
**a** MARS-MRI of the right hip, coronal plane, obtained 22 years post-operatively in a 72-year-old male patient. A cystic pseudotumor, measuring approximately 92 × 54 × 35 mm with a volume of about 90 mL, is observed adjacent to the joint. **b** MARS-MRI of the same right hip in an axial plane

A total of 19 hips in the study cohort fulfilled inclusion criteria for a MARS-MRI scan, and it was conducted for 18 hips (94.7%), identifying a pseudotumor in 3 of the 18 hips (16.7%) (Fig. 5). Comparative MRI data, gathered during the period 2013–2015, were available for 15 of 18 (83.3%) hips. Among them, 12 exhibited no suspicious findings in both MRI scans. A small pseudotumor identified in 2014, which was managed with “watchful waiting”, was absent on the current assessment. One patient lacked previous MRI data, while another patient with bilateral MoM THAs had shown a pseudotumor-like lesion only in the left hip a decade earlier and was now diagnosed with a pseudotumor formation in both hips. Revision surgery was recommended and performed in 2 of these 3 hips, mainly because of poor clinical function and severe osteolytic lesions.

### Metal ion analysis

After a minimum follow-up period of 20 years, blood metal ion levels were available for 51 unrevised hips (50%). Elevated metal ion levels (defined as cobalt or chromium levels > 2 μg/L) were detected in 11 out of 51 cases (21.6%). In 2 cases (3.9%), cobalt levels above 7 μg/L were documented (8.9 μg/L and 11.0 μg/L) while no chromium levels above this threshold were observed. In patients with elevated blood metal ions, a MARS-MRI was performed to rule out pseudotumor formation. One hip, demonstrating chromium levels of 1.0 µg/L and cobalt levels of 0.8 µg/L, was diagnosed with a pseudotumor formation with bone loss on MRI, necessitating revision surgery. The average chromium ion levels were 1.3 μg/L (range: 1.0–3.4 µg/L; SD 0.7 µg/L) and the average cobalt ion levels were 1.6 μg/L (range: 0.5–11.0 µg/L; SD: 2.2 µg/L). For all 51 hips, blood metal ion levels from a previous examination 10 years back were available. The current investigation revealed lower mean blood chromium levels (− 0.25 μg/L), although this was not statistically significant (*p* = 0.226). However, a significant increase in cobalt ion levels (+ 0.59 μg/L) was noted (*p* = 0.004). A clinically relevant increase in blood cobalt or chromium ion concentrations was observed in 11 hips (21.6%). A decline, mostly observed in chromium levels, was observed in 11 hips (21.6%).

## Discussion

Despite the frequent use of second-generation small-head MoM THAs between the years 1980 and 2010, longitudinal long-term data for these patients remain sparse. The survival rates for these implants reported in the literature vary, with predominantly quite positive outcomes [6–8]^.^ Andeol et al. reported a survival rate of 91% at 18 years [8], whereas Ishida et al. reported a survival rate of only 70% free of any revision at 20 years [6]. The latest National Joint Registry annual report for the year 2022 revealed a revision rate of 25% for uncemented THA with MoM bearings at 19 years, indicating that MoM bearings exhibit the worst survival rates among all bearing combinations in contemporary uncemented THA [2]. In line, revision in this study was most commonly (37%) indicated due to metal-wear-associated complications.

Nevertheless, the detected implant survival rate together with good functional results (mean reverse WOMAC score of 85) underline that second-generation small head MoM THAs achieve acceptable long-term results. The disparity in revision rates between the present study and the results reported in the literature is likely attributable to variations in mean follow-up time, as survival rates tend to decline more rapidly at the onset of the third decade after index surgery. In this study, the follow-up period of a minimum of 20 years and a mean of 23 years was longer than that of any registry data and most other studies. Another confounding factor may be the inconsistency in follow-up protocols, which are not standardized across studies, institutions, and countries. Additionally, if ARMD is detected, the differing management approaches employed could result in divergent outcomes.

Local adverse reactions to metal debris have been one of the main concerns after MoM. High pseudotumor rates of up to 72% at the beginning of the third decade post-surgery have been reported [15]. Yet, management of this condition remains a topic of debate. It has been discussed that asymptomatic patients with ARMD might not require revision surgery and may be successfully managed with close monitoring [15, 16]. Moon et al. reported on the natural history of pseudotumors after a follow-up of 15 and 20 years, respectively, demonstrating stable findings in 81% of patients and even a decrease in pseudotumor volume in one patient (4%) [15]. In the current cohort, 17% of the investigated hips exhibited a pseudotumor on MRI at a mean of 23 years after index surgery. Two out of the three hips underwent revision surgery due to poor clinical function and significant osteolysis. In contrast, one patient previously diagnosed with ARMD showed no abnormalities on the current MRI and demonstrated an excellent clinical function. This underscores the necessity for a nuanced approach and consideration of all pertinent information when deciding on revision surgery for patients with MoM.

The common occurrence of ARMD resulting in early failure and high revision rates in large-head (> 32 mm) MoM bearings [17] has led to a significant paradigm shift in the choice of bearing surfaces, resulting in the stop of the use of MoM implants around 10–15 years ago. Nonetheless, the clinical follow-up of these patients continues to play an important role in everyday clinical practice, and MoM is still utilized in hip resurfacing today. Patients with MoM bearings may require different follow-up protocols. These protocols typically encompass routine clinical evaluation, imaging studies (radiographs, ultrasound, MRI), and monitoring of systemic metal ion blood levels, with recommended follow-up intervals for asymptomatic patients of 3–5 years [13, 18–20]^.^

Routine radiographic follow-up revealed a concerning rate of osteolysis of 43% in our patient cohort, particularly around the proximal femur. This indicates inflammatory processes at the bone-implant interface due to wear debris and raises concerns for potential future revision surgery, as osteolysis may lead to aseptic loosening [21]. Jin et al. reported lower rates of periprosthetic osteolysis at 8% around the cup and 8% at the stem, though this was observed after a minimum follow-up period of only 15 years [22].

A major concern, alongside local adverse reactions, has been systemically elevated metal ion levels. Various cutoffs have been reported in the literature, warranting further investigation [13, 18–20]^.^ However, the clinical significance of elevated blood metal ions has not been clearly established in the past [23–25]. Many patients with elevated levels are asymptomatic, and the correlation with ARMD has been limited [26].

In our study, two patients presented with blood metal ion levels above 7 μg/L. Both patients had bilateral THAs with MoM bearings and had no clinical symptoms and good hip joint function based on the WOMAC score. Of these two patients, one declined further investigation with MARS-MRI or computed tomography (CT) due to claustrophobia, while the other showed no signs of ARMD on MARS-MRI.

On average, metal ion levels in our study were comparable to those reported in other long-term follow-up investigations [27, 28]. Compared to measurements taken 10 years previously from the same patient cohort, cobalt ion levels had shown a statistically significant mean increase (+ 0.59 μg/L; *p* = 0.004), while chromium levels had slightly decreased (− 0.25 μg/L; *p* = 0.226). These minor changes are presumably not clinically significant and most likely within the range of natural variation and the measurement error inherent to the different analytical methods employed.

A major limitation of this study is the incomplete data due to the long follow-up or patients being either unable or unwilling to attend an outpatient appointment. This resulted in the absence of radiographic follow-up for 35/102 (34%) patients and no collection of blood samples for 51/102 (50%) patients. This may have introduced bias into the data, potentially either under- or overreporting osteolysis rates and metal ion levels. However, these patients reported being free of any symptoms or issues related to the THA and had not undergone any prior revision surgery.

Moreover, the utilization of MARS-MRI was limited, potentially underestimating the pseudotumor rate, especially in asymptomatic patients. MRI examinations were performed only in patients with symptomatic hip replacements, previously documented pseudotumor formation, or high clinical suspicion of adverse reactions to metal debris (ARMD). In addition, a subset of patients declined MRI due to e. g., claustrophobia, long travel distances, absence of symptoms, or the perception that further imaging was unnecessary. As previously discussed, blood metal ion analysis alone may not be sufficient for the detection of ARMD. Additionally, computed tomography (CT) was utilized in only a small number of patients, potentially leading to an underestimation of the incidence of osteolysis, particularly in the acetabular region. Furthermore, the comparability of blood metal ion levels with other studies may be compromised due to documented variations in analytical methods, which have been shown to yield divergent results [29].

Another limitation of this study is the absence of a competing risk analysis (e.g., Fine–Gray model) despite the presence of two competing events (revision surgery and death). Therefore, the applied Kaplan–Meier analysis may have overestimated the probability of revision surgery due to death being treated as a censored event, and consequently may have underestimated long-term implant survivorship [30].

## Conclusions

Second-generation small-head metal-on-metal total hip arthroplasties demonstrate acceptable survival rates and functional outcomes more than 20 years after index surgery, but metal wear-related complications remain a significant concern, being the most common reason for revision surgery.

The observed high incidence of periprosthetic osteolysis (43%), elevated blood metal ion levels (22%), and metal wear–related complications (37% of revisions) raise significant safety concerns, thereby discouraging the use of small-head metal-on-metal bearings in current THA practice, while emphasizing the need for vigilant and long-term follow-up even in asymptomatic individuals.

## Data Availability

Data are available from the corresponding author upon reasonable request.

## Abbreviations

MoM: Metal-on-metal
THA: Total hip arthroplasty
WOMAC: Western Ontario and McMaster Universities Osteoarthritis Index
MARS-MRI: Magnetic resonance imaging with metal artifact reduction sequence
ARMD: Adverse reactions to metal debris
ICP-MS: Inductively coupled plasma-mass spectrometry
CT: Computed tomography
